# Short-term efficacy and safety of lurasidone versus placebo in antipsychotic-naïve versus previously treated adolescents with an acute exacerbation of schizophrenia

**DOI:** 10.1192/j.eurpsy.2022.11

**Published:** 2022-03-24

**Authors:** Christoph U. Correll, Michael Tocco, Jay Hsu, Robert Goldman, Andrei Pikalov

**Affiliations:** 1 Department of Psychiatry, Northwell Health, The Zucker Hillside Hospital, Glen Oaks, New York, USA; 2 Department of Psychiatry and Molecular Medicine, Hofstra Northwell School of Medicine, Hempstead, New York, USA; 3 Department of Child and Adolescent Psychiatry, Charité Universitätsmedizin, Berlin, Germany; 4 Global Medical Affairs, Sunovion Pharmaceuticals Inc., Fort Lee, New Jersey, USA; 5 Sunovion Pharmaceuticals Inc., Marlborough, Massachusetts, USA

**Keywords:** Atypical antipsychotic, controlled clinical trial, lurasidone, schizophrenia, second generation antipsychotic

## Abstract

**Background:**

To evaluate the efficacy of short-term lurasidone in antipsychotic treatment-naïve (TN) adolescents with schizophrenia versus those treated previously (TP) with antipsychotics.

**Methods:**

Patients aged 13–17 with schizophrenia, and a Positive and Negative Symptom Scale (PANSS) score ≥ 70 and < 120, were randomized to 6 weeks of double-blind treatment with lurasidone (40 or 80 mg/day) or placebo. In a post-hoc, pooled-dose analysis, efficacy was evaluated for TN (criteria: never received antipsychotic treatment) versus TP at the time of the study. Treatment response criteria: ≥20% reduction in PANSS total score.

**Results:**

Altogether, 57 TN and 269 TP patients enrolled in the 6-week DB study. Mean endpoint change in PANSS total score was significantly greater for lurasidone versus placebo in both the TN group (−25.0 vs. -14.4; *p* < 0.02; effect size = 0.75), and in the TP group (−17.3 vs. -10.0; *p* < 0.001; effect size = 0.45); and responder rates were higher for lurasidone versus placebo in both the TN group 84.6% versus 38.9%; number needed to treat [NNT] = 3 and in the TP group (60% vs. 42%; NNT = 6). Rates of treatment-emergent adverse events, and mean changes in body weight and metabolic parameters were similar for the TN and TP groups.

**Conclusions:**

In a 6-week, placebo-controlled trial, lurasidone demonstrated significant efficacy in adolescents with schizophrenia regardless of previous antipsychotic therapy status; however, the effect size was notably larger in the TN patient group. In both the TN and TP groups, minimal effects were noted on weight, metabolic parameters, or prolactin.

## Introduction

Schizophrenia is a debilitating neurodevelopmental disorder that typically emerges in late adolescence and early adulthood [1]. The disorder is estimated to affect approximately 21 million people worldwide and is characterized by substantial ongoing disability [2, 3]. The lifespan of individuals with schizophrenia is estimated to be 14.5 years shorter on average compared to the general population due to various factors, including notably higher rates of tobacco smoking, alcohol/drug use, cardiovascular disease, diabetes, and poor dietary habits [4–10].

Meta-analytic evidence indicates that a younger age at onset of schizophrenia is associated with more hospitalizations, more negative symptoms, more relapses, and poorer social/occupational functioning and global outcome [11]. However, there is also some conflicting evidence indicating that factors other than earlier age of onset of schizophrenia determine poorer outcomes [12]. Outcome predictors aside, it is especially important to establish the clinical efficacy and safety of antipsychotic therapies during the developmentally sensitive biopsychosocial phase of adolescence. Although atypical antipsychotics are recommended first-line treatments for schizophrenia, concerns about weight gain, risk of diabetes, and metabolic problems associated with many of the approved atypical antipsychotics is an important public health concern [13–16]. These concerns are particularly relevant for children and adolescents where weight gain and the risk of developing hyperglycemia, hyperlipidemia, hyperprolactinemia, and diabetes with atypical antipsychotics has been extensively documented [17–21].

Given the typical onset of illness, many adolescents with a diagnosis of schizophrenia may be experiencing their first episode and have never received antipsychotic medication. Not receiving antipsychotic medication for an extended period after diagnosis of psychosis has been associated with structural changes in the brain and relatively poor clinical outcomes [22–27]. Several studies have examined the efficacy of different antipsychotics among first-episode and treatment-naïve (TN) adult patients [28–35]. However, whether or not these findings generalize to an adolescent population is uncertain. For example, there is some evidence that TN adolescent onset schizophrenia may have a different pattern of cortical gray matter deficits compared with TN adult onset schizophrenia [36,37]. Drug response of TN adolescents with schizophrenia (or previously treated) may therefore differ from TN adults with schizophrenia.

The atypical antipsychotic agent lurasidone shows high binding affinity for D_2_, 5-HT_2A_, and 5-HT_7_ receptors (antagonist); moderate affinity for 5-HT_1A_ receptors (partial agonist); and no appreciable affinity for H_1_ and M_1_ receptors [38]. Efficacy and safety of lurasidone in the acute and long-term treatment of adults with schizophrenia in the dose range of 40–160 mg/day has been demonstrated across multiple studies [39–46]. A low propensity for weight gain and metabolic disturbance was consistently found across these lurasidone trials [47,48]. The absence of activity for lurasidone at 5HT_2C_ and histamine H_1_ receptors is thought to be responsible for the minimal observed effect on weight [49–51]. In a previously reported 6-week, double-blind, placebo-controlled, fixed-dose trial, treatment with 40 and 80 mg/day doses of lurasidone were found to be safe and generally well-tolerated, and to have significant efficacy in adolescents with schizophrenia [52]. Based on the efficacy and safety results from this short-term study, the results of a pharmacokinetic study [53], and a subsequent long-term study in adolescents [54], lurasidone has been approved, in doses of 40–80 mg/day, by the U.S. Food and Drug Administration for the treatment of schizophrenia in youth 13–17 years old.

Few studies have examined treatment response in adolescents with schizophrenia who are TN compared to previously treated adolescents; and no study (to the best of our knowledge) has examined this question in the context of a placebo-controlled study, either in adolescents or in adults. The objective of the current post-hoc analysis was to evaluate the short-term efficacy and safety of lurasidone in TN adolescent patients with a diagnosis of schizophrenia compared with a previously treated group.

Based on previous data indicating that first-episode and TN patients generally respond better to antipsychotics than patients who have been ill for longer [26, 27, 55], but are also more sensitive to adverse effects of antipsychotics [19–21], we hypothesized that short-term treatment with lurasidone in antipsychotic-naïve patients would be associated with greater efficacy and adverse effect burden (relative to placebo) than in previously treated adolescents.

## Methods

The study used in the current post-hoc analysis was a double-blind (DB), parallel-group, placebo-controlled, multicenter trial that randomized patients (1:1:1) to 6 weeks of fixed-dose treatment with lurasidone (40 or 80 mg/day) or placebo. Eligible patients were aged 13–17 years with a Diagnostic and Statistical Manual of Mental Disorders, Fourth Edition (Text Revision; DSM-IV-TR) diagnosis of schizophrenia who were experiencing an acute exacerbation (≤2 months in duration) of symptoms defined by a Positive and Negative Syndrome Scale (PANSS) [56] total score ≥70 and a Clinical Global Impression-Severity (CGI-S) [57] score ≥ 4 (at least moderately ill). Patients were excluded if they had a history of intellectual disability or any neurologic disorder; or an alcohol or substance use disorder diagnosis in the previous 6 months. Additional details on study design and study entry criteria may be found in the primary publication [52].

The study protocol and any amendments were reviewed and approved by institutional review boards at each investigational site. Written informed consent was obtained from a parent or legal guardian, and assent was obtained from each adolescent patient prior to the conduct of any study procedures.

### Concomitant medication

Concomitant treatment with antidepressants and stimulants (for ADHD) was permitted. Concomitant use of lorazepam or equivalent benzodiazepine was permitted at the discretion of the investigator (≤6 mg/day or equivalent dose) for intolerable anxiety/agitation. Benzodiazepine and nonbenzodiazepine sedative-hypnotic agents were also permitted on an as-needed basis for insomnia. Treatment with benztropine (≤6 mg/day) or alternative medications was permitted as needed for movement disorders and treatment with propranolol (≤120 mg/day) was permitted as needed for akathisia. However, prophylactic use of medications to treat movement disorders was not permitted.

### Study assessments

#### Efficacy

Efficacy measures included the PANSS total score and the PANSS Positive and Negative subscales [56]; the CGI-S; the clinician-rated Children’s Global Assessment Scale (CGAS) [58]; and the Pediatric Quality of Life Enjoyment and Satisfaction Questionnaire (PQ-LES-Q) [59]. The CGAS is a clinician-administered measure that evaluates global impairment on a scale of 0–100, with higher scores indicating better outcomes. The PQ-LES-Q is a quality of life measure that has demonstrated reliability and validity in youth [59]. Efficacy and safety assessments were performed by trained site-based raters. Prior treatment status was obtained from self-report by caregiver and patient in response to standard questions during the screening visit for the DB study.

#### Safety and tolerability assessments

Adverse events were recorded based on spontaneous report and also by administration of the Udvalg for Kliniske Undersogelser (UKU) Side Effect Rating Scale, a clinician-rated scale consisting of 48 adverse events divided into four categories (psychic, neurologic, autonomic, and other) [60]. Mean severity scores (1—mild to 4—severe) were calculated for the total score and each side effect category. Movement disorders were assessed by three scales: the Simpson–Angus Scale (SAS) [61], the Barnes Akathisia Rating Scale (BARS) [62], and the Abnormal Involuntary Movement Scale (AIMS) [57, 63]. Suicidal ideation and behavior were measured with the Columbia Suicide Severity Rating Scale (C-SSRS) [64]. Additional safety evaluations included vital signs, weight, laboratory tests (metabolic parameters and other blood chemistry and hematology parameters), 12-lead electrocardiogram (ECG), and physical examination.

### Statistical analyses

The efficacy population was defined as all patients enrolled who received at least one dose of study medication in the trial and had at least one postbaseline efficacy assessment. Efficacy measures were examined in terms of least squared mean (SD) change from DB study baseline to week 6 or last observation carried forward (LOCF) endpoint, depending on statistical methodology used. Efficacy at week 6 (or LOCF endpoint) was compared for lurasidone (both doses combined) versus placebo in two patient subgroups based on prior treatment status: TN versus previously treated. TN was defined as never having been treated with an antipsychotic medication prior to study entry; and patients in the previously treated subgroup had been treated with an antipsychotic medication prior to study entry. Note that the data on the number and adequacy of previous antipsychotic medication trials was not available.

Change scores were analyzed using a mixed model for repeated measurement (MMRM) analysis or analysis of covariance (ANCOVA) using an LOCF approach. Effect sizes (ES) were calculated as the least squares mean difference in week 6 (or endpoint) change score for lurasidone versus placebo divided by the pooled standard deviation. Standardized mean difference in endpoint change scores (lurasidone vs. placebo) were also calculated for the TN versus previously treated groups. Treatment response was calculated, based on LOCF-endpoint data, using both ≥20% and ≥50% improvement from double-blind baseline in PANSS total score. The number-needed-to-treat (NNT) was calculated as the reciprocal of the difference in response rates for lurasidone versus placebo at LOCF-endpoint. All significance tests were two-tailed with alpha = 0.05. Baseline differences in the demographic and clinical characteristics of the TN and previously treated groups (for the combined study treatments) were analyzed using Fishers’ exact test (for sex and race), two-sample *t*-tests (age, age of onset, duration of current episode, PANSS total score, CGI-S score, CGAS score, and Q-LES-Q score), and chi-square (prior hospitalization).

The safety population was defined as all patients who were enrolled who received at least one dose of lurasidone in that study. Safety analyses were descriptive and included the number (%) of treatment-emergent adverse events, discontinuations due to adverse events, and use of medications for acute extrapyramidal symptoms. Means with 95% confidence intervals (CIs), or NNH were reported as appropriate. Observed case analyses were calculated for change from double-blind baseline for safety variables, including weight, laboratory tests, ECG parameters (including QTcF, Fridericia’s formula), and movement disorder scale scores (SAS, BARS, AIMS).

## Results

### Patient disposition and characteristics

**Figure 1.**
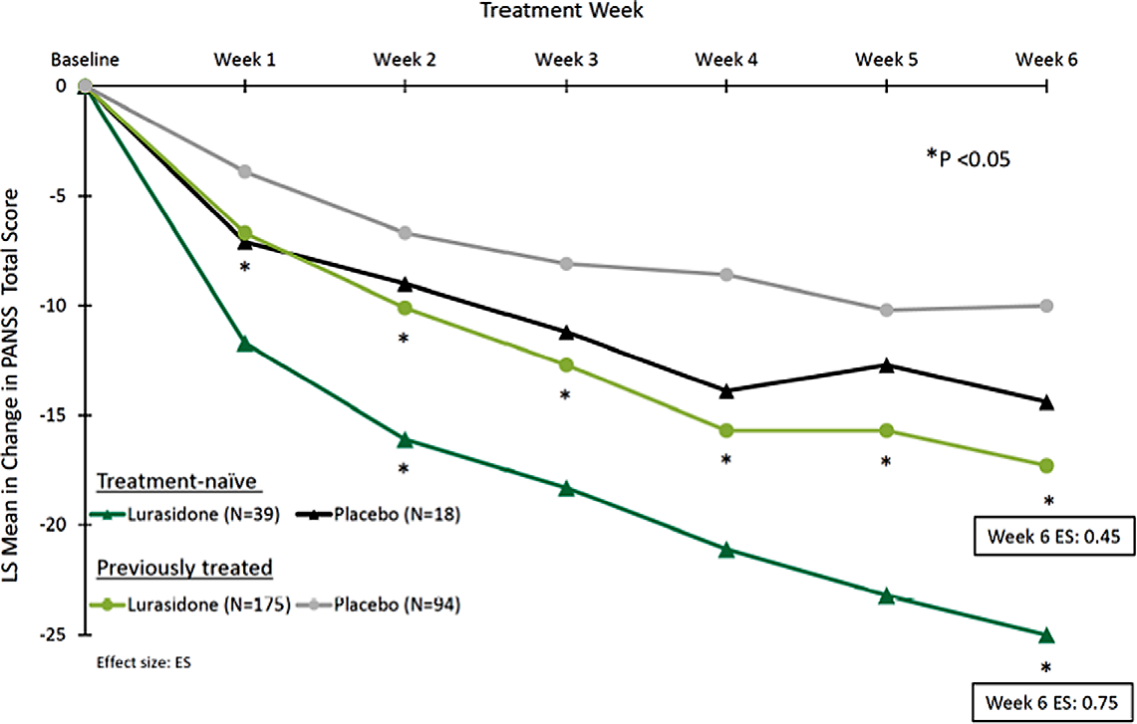
Change from double-blind baseline in PANSS total score.

At DB baseline, there were 57 TN patients and 269 previously treated patients (Figure 1). Of these, 50 (87.7%) TN and 221 (82.2%) previously treated patients completed the 6-week DB study.

Table 1 presents demographic and clinical characteristics of patients at DB baseline, separately for the TN group and previously treated group. In general, the baseline characteristics of the TN and previously treated groups were similar, with the following notable exceptions: (a) the proportion of patients with prior hospitalizations for schizophrenia was 58% in the previous treatment group and 30% in the TN group (*p* < 0.001); (b) the CGAS score was statistically significantly lower (*p* < 0.001; more functional impairment) in the previous treatment group compared to the TN group; and (c) the CGI-Severity score was higher in the previous treatment group compared to the TN group. It should be noted that the between-group differences in the CGAS and CGI-S, while statistically significant were of modest clinical significance.

**Table 1.** Patient characteristics at double-blind baseline.

	Treatment-naïve		Previously treated	
Characteristic	Lurasidone (*N* = 39)	Placebo (*N* = 18)	Lurasidone (*N* = 175)	Placebo (*N* = 94)
Male, *n* (%)	24 (61.5)	13 (72.2)	113 (64.6)	58 (61.7)
White, *n* (%)	26 (66.7)	11 (61.1)	120 (68.6)	63 (67.0)
Age, years, mean (SD)	15.2 (1.5)	14.9 (1.4)	15.4 (1.3)	15.4 (1.4)
Age of onset of psychotic symptoms, years, mean (SD)	13.5 (2.5)	14.0 (1.8)	13.0 (2.9)	12.9 (2.8)
Previous hospitalizations for schizophrenia ≥ 1, *n* (%)**	12 (30.8)	5 (27.8)	100 (57.1)	56 (59.6)
Duration of current psychotic episode, weeks, mean (SD)	5.5 (8.3)	6.7 (9.3)	7.3 (18.3)	6.1 (18.6)
PANSS total score, mean (SD)	92.7 (9.5)	89.2 (8.9)	94.6 (11.3)	93.5 (11.4)
CGI-S, mean (SD)*	4.8 (0.7)	4.4 (0.6)	4.9 (0.6)	4.8 (0.6)
CGAS, mean (SD)**	48.2 (8.9)	47.4 (9.0)	43.6 (8.5)	43.3 (8.1)
PQ-LES-Q, mean (SD)^a^	54.9 (16.7)	52.9 (10.6)	51.9 (18.1)	52.4 (16.5)

*Note*: Significance testing of between-group difference in baseline characteristics for previously treated versus previously treated groups.

Abbreviations: CGAS, clinical global assessment scale; CGI-S, clinical global impression-severity; PANSS, positive and negative syndrome scale; PQ-LES-Q, pediatric quality of life, enjoyment and satisfaction questionnaire; SD, standard deviation.

*P < 0.05: CGI-S; **P < 0.001. Prior hospitalization and CGAS.

a Mean percentage of maximum possible score.

### Efficacy for TN and previously treated patients

Compared to placebo, treatment with lurasidone was associated with significantly greater improvement from DB baseline to week 6 (MMRM) in the PANSS total score for both the TN (*p* = 0.016; ES = 0.75) and previously treated groups (*p* = 0.0008; ES = 0.45; Table 2). Within the TN group, there was significantly greater improvement for lurasidone, compared to placebo on the PANSS Positive Symptom score (*p* = 0.0045; ES = 0.89), but not the PANSS Negative Symptom score (*p* = 0.18; ES = 0.40; Table 2). Within the previously treated group, treatment with lurasidone was associated with greater improvement versus placebo in the PANSS Positive Symptom score (*p* < 0.0001; ES = 0.57) and in the Negative Symptoms score (*p* = 0.017; ES = 0.32) at week 6 (MMRM; Table 2). Among TN patients, the proportion of lurasidone versus placebo responders was 84.6% versus 38.9% (NNT = 3) based on the ≥ 20% PANNS improvement criterion, and 30.8% versus 8.5% (NNT = 7) based on the ≥ 50% PANNS improvement criterion. Among previously treated patients, the proportion of lurasidone versus placebo responders was 60.0% versus 42.6% (NNT = 6) based on the ≥20% PANNS improvement criterion, and 18.9% versus 8.5% (NNT = 10) based on the ≥50% PANNS improvement criterion.

**Table 2.** Mean (SE) change from DB baseline on efficacy measures.

Efficacy measure	Treatment-naive			Previously treated		
	Lurasidone (*N* = 39)	Placebo (*N* = 18)	Effect Size	Lurasidone (*N* = 175)	Placebo (*N* = 94)	Effect size
PANSS total (SE)	−25.0 (2.5)*	−14.4 (3.6)	0.75	−17.3 (1.3)***	−10.0 (1.8)	0.45
PANSS positive (SE)	−8.8 (0.9)**	−4.5 (1.3)	0.89	−5.9 (0.4)***	−3.1 (0.6)	0.57
PANSS negative (SE)	−4.8 (0.8)	−3.0 (1.2)	0.40	−3.7* (0.4)*	−2.2 (0.5)	0.32
CGI-S (SE)	−1.07 (0.15)**	−0.28 (0.2)	0.97	−0.91 (0.08)**	−0.55 (0.11)	0.38
CGAS (SE)	8.5 (2.4)	2.7 (2.9)	0.55	11.1 (1.0)**	6.5 (1.2)	0.43
PQ-LES-Q^a^ (SE)	2.0 (2.3)	−3.5 (2.8)	0.54	6.3 (1.1)***	0.7 (1.4)	0.47

*Note*: For PANSS scales and CGI-S scores are estimated least square mean change to week 6 derived from mixed model for repeated measurements. For CGAS and PQ-LES-Q, scores are change from baseline to Week 6 LOCF endpoint derived from ANCOVA analyses.

Abbreviations: CGAS, clinical global assessment scale; CGI-S, clinical global impression-severity; PANSS, positive and negative syndrome scale; PQ-LES-Q, pediatric quality of life, enjoyment and satisfaction questionnaire; SD, standard deviation; SE, standard error.

*p < 0.05; **p < 0.01; ***p < 0.001, in comparison to placebo.

a Mean percentage of maximum possible score.

Significantly greater improvement on the CGI-Severity scale was also observed for lurasidone-treated patients compared to placebo in both the TN group (*p* = 0.0023; ES = 0.97) and previously treated group (*p* = 0.005; ES = 0.38; Table 2). Lurasidone treatment (vs. placebo) was associated with slightly larger endpoint effect sizes in the TN group compared to the previously treated group on the Children’s Global Assessment Scale (ES = 0.55 vs. 0.43) and on the PQ-LES-Q (ES = 0.54 vs. 0.47), though significant differences from placebo were not obtained for the TN group due to reduced statistical power as a result of smaller sample size (Table 2). With the larger sample size in the previously treated group, significantly greater improvement for lurasidone, compared to placebo, was obtained on both the CGAS (*p* = 0.0012) and PQ-LES-Q (*p* = 0.0004).

### Safety and tolerability for TN and previously treated patients

The rates of TEAEs in lurasidone-treated patients were similar for the TN group and the previously treated groups (Table 3). Within the TN group, lurasidone-treated patients reported nausea, anxiety, akathisia, vomiting, and somnolence at 10% or greater incidence and rates twice (or greater) than that found in the placebo group. In the previously treated group, lurasidone-treated patients reported nausea, akathisia, and vomiting at 10% incidence and rates twice (or greater) than that found among placebo-treated patients. Within the TN group, the incidence of extrapyramidal symptom-related adverse events (excluding akathisia) was 5.1% (*n* = 2) in the lurasidone group and 0% in the placebo group (Table 3). Among those that were not TN, 9 (5.1%) lurasidone-treated patients and 2 (2.1%) placebo-treated patients reported extrapyramidal symptom-related adverse events (excluding akathisia).

**Table 3.** Adverse events during 6-weeks of double-blind, placebo-controlled treatment.

Adverse effect (*n*, %)	Treatment-naïve			Previously treated		
	Placebo (*N* = 18)	Lurasidone (*N* = 39)	NNH	Placebo (*N* = 94)	Lurasidone (*N* = 175)	NNH
Any TEAE	6 (33.3)	31 (79.5)		58 (61.7)	106 (60.6)	
Nausea	1 (5.6)	5 (12.8)	14	2 (2.1)	24 (13.7)	9
Anxiety	0	5 (12.8)	8	9 (9.6)	9 (5.1)	NA
Somnolence	0	4 (10.3)	10	6 (6.4)	18 (10.3)	26
Nasopharyngitis	0	2 (5.1)	20	5 (5.3)	7 (4.0)	NA
Akathisia	0	5 (12.8)	8	2 (2.1)	14 (8.0)	17
Agitation	1 (5.6)	1 (2.6)	NA	4 (4.3)	10 (5.7)	72
Dry mouth	0	2 (5.1)	20	0	3 (1.7)	59
Pyrexia	0	2 (5.1)	20	1 (1.1)	1 (0.6)	NA
Hypersomnia	0	2 (5.1)	20	0	0	NA
Sedation	0	3 (7.7)	13	2 (2.1)	5 (2.9)	125
Pain in extremity	0	2 (5.1)	20	1 (1.1)	1 (0.6)	NA
Dizziness	1 (5.6)	2 (5.1)	NA	0	8 (4.6)	22
Vomiting	1 (5.6)	5 (12.8)	14	1 (1.1)	11 (6.3)	20
Diarrhea	0	3 (7.7)	13	1 (1.1)	5 (2.9)	56
Extrapyramidal related TEAEs (excluding akathisia)	0	2 (5.1)	20	2 (2.1)	9 (5.1)	34

Treatment-emergent adverse events (≥ 5% on lurasidone and greater than placebo).

Abbreviations: NNH, number-needed-to-harm; TEAE, treatment-emergent adverse event.

On the UKU, change from baseline to endpoint in the four side effect categories (psychic, neurologic, autonomic, and other) was generally similar between the pooled lurasidone dose groups and the placebo group for the TN group, and also for the previously treated group (Table 4). Within the TN group, the proportion of patients with treatment-emergent suicidal ideation, measured by the C-SSRS, was 7.7% (*n* = 3) in the lurasidone group and 5.6% (*n* = 1) in the placebo group. For the previously treated group, 2.3% (*n* = 4) of patients were found to have treatment-emergent suicidal ideation on the CSSR in the lurasidone group compared to 4.3% (*n* = 4) in the placebo group. No occurrence of suicidal behavior or completed suicide was evident in either the TN or previously treated groups.

**Table 4.** Udvalgfor Kliniske Undersogelser Side Effect Rating Scale Scores:Mean (SD) Baseline-to-EndpointChange.

	Treatment-naïve		Previously treated	
UKU adverse effect ratings	Placebo (*N* = 18)	Lurasidone (*N* = 39)	Placebo (*N* = 94)	Lurasidone (*N* = 175)
Psychic side effects (SD)	−0.3 (2.8)	−1.8 (3.3)	−1.0 (3.4)	−1.1 (3.0)
Neurologic side effects (SD)	0.0 (0.4)	0.1 (0.7)	−0.1 (0.7)	0.0 (0.8)
Autonomic side effects (SD)	0.0 (0.0)	−0.3 (1.2)	−0.1 (1.1)	0.0 (0.8)
Other side effects (SD)	0.1 (0.4)	−0.1 (1.2)	0.1 (0.9)	0.1 (0.8)
UKU total score (SD)	−0.3 (2.8)	−2.1 (4.8)	−1.1 (4.1)	−1.1 (3.7)

*Note*: Higher scores indicate greater severity; range of 0–30 for psychic, 0–24 for neurologic, 0–33 f for autonomic, and 0–48 for other. Udvalg for Kliniske Undersogelser side effect rating scale scores: mean (SD) baseline-to-endpoint change.

Abbreviations: SD, standard deviation; UKU, Udvalg for Kliniske Undersogelser.

Among TN patients, the proportion who received anticholinergic medications for acute extrapyramidal symptoms in the lurasidone versus placebo groups were 2.6% versus 0%; and the proportion who received benzodiazepines were 17.9% versus 16.7%. Among previously treated patients, the proportion who received anticholinergic medications for acute extrapyramidal symptoms in the lurasidone versus placebo groups were 4.0% versus 2.1%; and the proportion who received benzodiazepines were 21.1% versus 24.5%).

No clinically meaningful mean changes from baseline to endpoint were evident in the TN group for lurasidone or placebo, respectively, for the SAS (0.03 vs. 0.02), BARS (0.4 vs. 0.1), and AIMS (0.3 vs. 0.1). Similarly, no clinically meaningful mean changes from baseline to endpoint were evident in the previously treated group for lurasidone or placebo for the SAS (0.01 vs. 0.01), BARS (0.0 vs. 0.0), and AIMS (0.0 vs. 0.0).

Laboratory measures of lipid parameters, glycemic indices, and prolactin levels also showed no clinically meaningful differences between lurasidone and placebo for the TN and for the previously treatment group (Table 5). Within the TN group, there was one patient in the lurasidone group, and none in the placebo group, that experienced a clinically meaningful (≥ 7%) increase in body weight. Within the previously treated group, there were 8 (4.6%) patients in the lurasidone group and 9 (9.6%) in the placebo group with clinically meaningful weight change. No patients had a QTcF ≥ 460 milliseconds or a postbaseline change in QTcF ≥ 60 ms in either the TN group or the previously treated group.

**Table 5.** Change from double-blind baseline in laboratory values and weight/BMI.

	Treatment-naïve		Previously treated	
Cardiometabolic outcomes	Placebo (*N* = 18)	Lurasidone (*N* = 39)	Placebo (*N* = 94)	Lurasidone (*N* = 175)
Metabolic labs, mg/dL, mean (95% CI)				
Total cholesterol	−18.1 (−31.6, −4.7)	+1.3 (−10.4, 13.1)	−6.3 (−11.3, −1.4)	−2.8 (−6.8, 1.2)
LDL cholesterol	−13.6 (−25.0, −2.1)	−1.2 (−10.5, 8.0)	−3.5 (−7.5, 0.5)	−2.0 (−5.4, 1.4)
Triglycerides	+3.2 (−18.2, 24.6)	−8.7 (−34.4, 17.0)	+0.4 (−10.9, 11.7)	+3.6 (−5.0, 12.2)
Glucose	−3.3 (−13.7, 7.2)	−0.1 (−5.1, 4.9)	+0.3 (−2.5, 3.1)	+0.3 (−1.3, 2.0)
Hemoglobin A1C (%), mean (95% CI)	−0.05 (−0.15, 0.04)	−0.01 (−0.07, 0.04)	+0.02 (−0.03, 0.06)	+0.01 (−0.03, 0.05)
Insulin (mU/L), mean (95% CI)	−1.8 (−32.2, 28.6)	−1.9 (−12.5, 8.7)	−3.9 (−15.2, 7.4)	−0.7 (−7.1, 5.6)
Prolactin, (ng/mL), mean (95% CI)				
Female	+9.6 (−19.1, 38.3)	+4.8 (−1.1, 10.8)	−4.0 (−11.9, 3.8)	+4.8 (−3.2, 12.8)
Male	−1.2 (−4.2, 1.9)	0.3 (−3.1, 3.7)	+0.4 (−6.3, 7.0)	+1.2 (−1.6, 4.0)
Body weight, kg, mean (95% CI)	0.41 (−0.19, 1.01)	0.36 (−0.15, 0.87)	0.17 (−0.40, 0.73)	0.51 (0.21, 0.81)
Effect size (vs. placebo)		−0.04		0.14

## Discussion

To the best of our knowledge, this is the first placebo-controlled study that has examined the efficacy and safety of an atypical antipsychotic in TN adolescents (or adults) with schizophrenia. The results of this post-hoc analysis suggest that lurasidone is an effective treatment option for both antipsychotic-naïve adolescents diagnosed with schizophrenia and adolescents previously treated with antipsychotic medication. The magnitude of the treatment effect (lurasidone vs. placebo), measured by effect sizes at endpoint, was greater for TN versus previously treated patients on the PANSS total score (ES = 0.75 vs. 0.45). Similarly, the standardized mean difference score was also greater for TN versus previously treated patients on the PANSS total score. These results compare favorably to results of a previous meta-analysis of acute antipsychotic treatment of adolescents with schizophrenia-spectrum disorders, where the standard mean difference (SMD) scores ranged from −0.34/−0.38 (aripiprazole/asenapine) up to −0.57/−0.59 (risperidone/olanzapine) [65]. In addition, in a network meta-analysis comparing atypical antipsychotics in the treatment of adolescent schizophrenia [66], treatment with lurasidone had comparable efficacy to other atypical antipsychotics, but lower risk of all-cause discontinuation than aripiprazole and paliperidone and lower risk of weight gain than all other included atypical antipsychotics, except for aripiprazole and ziprasidone.

Currently, regulatory approval of lurasidone in the United States for adolescents with schizophrenia is limited to doses of 40–80 mg/day. However, the results of a recent analysis [67] of young adults (ages 18–25) with schizophrenia reported that use of higher doses (120–160 mg/day) is associated with a notable increase in effect size. For example, the effect size of lurasidone 80 mg/day (vs. placebo) on the PANSS total score increased from 0.57 to 0.90 for lurasidone 120–180 mg/day. It is possible that lower effect sizes observed in the current study in the previously treated adolescent patient group may be largely dose-related and respond to use of higher doses.

Lurasidone was generally well-tolerated in both TN and previously treated adolescents with schizophrenia. Notably, in the TN group, only two AEs occurred with an NNH < 10 (anxiety, NNH = 8; akathisia, NNH = 8), and only one AE in the previously treatment group (nausea, NNH = 9). This relatively favorable tolerability profile in TN patients, who are generally more sensitive to adverse effects, especially weight gain [18–20], confirms the recent finding from a large systematic review that found lurasidone to be the most well-tolerated antipsychotic when comparing adverse effects versus placebo in pediatric patients [68].

Given that the current results are based on post-hoc analyses with limited sample sizes, additional data from prospective studies are needed before any firm conclusions may be made. However, the finding that the efficacy of lurasidone among TN patients is at least as strong, if not stronger, than that found for previously treated patients has potentially important implications. For one, these results suggest that antipsychotic efficacy among TN adults [28–35, 69] may extend to TN adolescents, despite research indicating that the extent and pattern of gray matter deficits in early onset schizophrenia may be different from the deficits observed in adult-onset schizophrenia [36, 37]. Further prospective placebo-controlled studies of lurasidone and other antipsychotics are needed to confirm efficacy among TN adolescents with schizophrenia.

A second potential implication of the current findings is that TN adolescent patients appear to respond especially well to early treatment intervention with lurasidone, while *not* receiving early treatment may result in a relatively poor clinical outcome that may include a greater degree of brain structural changes [22–25]. Shortening the duration of untreated psychosis by earlier antipsychotic intervention is likely to improve outcomes in youth with early-onset schizophrenia [70, 71].

Alternatively, one might hypothesize that the relatively greater treatment effect observed for the TN versus previously treated group (effect size: 0.75 vs. 0.45) is largely attributable to a reduced response rate in the previously treated group. This relative treatment resistance might be due to the progressive nature of the schizophrenic illness since chronicity is a well-established predictor of reduced antipsychotic response [72]. It is also possible that the potency of antipsychotic drugs is diminished across repeated courses of treatment, though this treatment effect might be difficult to disentangle from the effects of chronicity.

In the current study, lurasidone was found to have minimal effects on weight, prolactin, and metabolic measures, even in the TN subgroup, although with other atypical antipsychotics treatment-naïveté has previously been shown to be a strong risk factor for enhanced cardiovascular risk [19]. Since adolescents with a diagnosis of schizophrenia (vs. non-schizophrenia controls) are an especially high-risk population, with a long-term outcome characterized by increased cardiovascular morbidity and mortality, and a significantly shorter life span (~20 years) [15, 73], choice of antipsychotic should include consideration of metabolic adverse effects [21, 74, 75].

Several limitations of this study should be noted. First, the comparisons of TN and previously treated patients were based on post-hoc analyses, and thus should be considered exploratory. Second, the inclusion and exclusion criteria may limit the generalizability of these results. Third, the 6-week duration of the study does not allow for a comparison versus placebo over longer periods if time when, for example, body weight changes might accumulate. However, antipsychotic-related weight gain generally occurs early and predicts later weight gain [19, 76]. Furthermore, longer exposure to placebo is ethically questionable. Also, it should be noted that data in 271 patients who entered the 2-year open-label extension study of the current randomized trial found that efficacy advantages in the TN (vs. previously treated) population extended into the long-term treatment phase and that the tolerability was also maintained in both patient groups [54]. Fourth, the sample size of the antipsychotic-naïve adolescent group was relatively modest; however, to the best of our knowledge, this is the first sample of antipsychotic-naïve patients with schizophrenia, adolescent or adult, that has been part of a placebo in a double blind, placebo-controlled trial of an antipsychotic. A final limitation of the current post-hoc analysis was that medical records were not obtained for patients entering the current clinical trial. Consequently, we do not have reliable information on adequacy of previous treatment (dosing or duration) or response to previous treatment. Additional studies that more fully characterize the previous treatment history are needed to evaluate the efficacy and safety of lurasidone and other antipsychotics among TN and previously treated adolescents with schizophrenia.

In conclusion, the current post-hoc analysis found lurasidone (40 and 80 mg/day) to be a safe, well-tolerated, and effective short-term treatment in adolescents with schizophrenia, regardless of prior treatment history. However, notably larger treatment effect sizes were observed in the subgroup of patients who had never received antipsychotic therapy compared to patients with previous antipsychotic exposure. Consistent with previously reported safety findings in both adult and pediatric populations, no clinically meaningful changes in weight, metabolic parameters, and prolactin were evident with lurasidone treatment. However, in contrast to our hypothesis based on results from studies of other antipsychotics, favorable tolerability was also observed for lurasidone in the TN subgroup, despite previous findings that this subgroup is generally more sensitive and adverse effect prone. This favorable short-term benefit–risk profile suggests that lurasidone should be considered as a potential first-line treatment for the acute management of adolescents with schizophrenia. Complementary and consistent data from the long-term open-label extension study of this trial indicate that the first-line status for lurasidone extends from the acute into the longer-term management of adolescents with schizophrenia.

## Data Availability

Data are available upon request from Dr. Michael Tocco (michael.tocco@sunovion.com).
